# The Role of N^6^-Methyladenosine in Inflammatory Diseases

**DOI:** 10.1155/2022/9744771

**Published:** 2022-12-12

**Authors:** Haojun Xu, Changjie Lin, Jinghan Yang, Xi Chen, Yingyu Chen, Jianguo Chen, Aizhen Guo, Changmin Hu

**Affiliations:** ^1^Department of Clinical Veterinary Medicine, College of Veterinary Medicine, Huazhong Agricultural University, Wuhan, China; ^2^State Key Laboratory of Agricultural Microbiology, Huazhong Agricultural University, Wuhan, China; ^3^Department of Preventive Veterinary Medicine, College of Veterinary Medicine, Huazhong Agricultural University, Wuhan, China; ^4^Hubei Hongshan Laboratory, Huazhong Agricultural University, Wuhan, China

## Abstract

N^6^-Methyladenosine (m^6^A) is the most abundant epigenetic RNA modification in eukaryotes, regulating RNA metabolism (export, stability, translation, and decay) in cells through changes in the activity of writers, erasers, and readers and ultimately affecting human life or disease processes. Inflammation is a response to infection and injury in various diseases and has therefore attracted significant attention. Currently, extensive evidence indicates that m^6^A plays an essential role in inflammation. In this review, we focus on the mechanisms of m^6^A in inflammatory autoimmune diseases, metabolic disorder, cardio-cerebrovascular diseases, cancer, and pathogen-induced inflammation, as well as its possible role as targets for clinical diagnosis and treatment.

## 1. Introduction

With the increasing RNA modification mapping technique, hundreds of modifications have been found in RNA that, taken together, represent the posttranscriptional regulatory mechanisms. RNA modifications are responsible for regulating gene translation in a precise and detailed spatiotemporal manner. Among them, significant attention has been focused on methylation modification. N^6^-Methyladenosine (m^6^A) chemical modifications involve addition of an extra methyl to the adenylate 6-position N atom in a specific sequence (i.e., RRACH), and they are widely found distributed in mRNA and ncRNA [1]. m^6^A is the most abundant RNA modification in mammalian cells [2–4]. It is estimated that about 0.1%–0.4% of the adenosine in RNA carries an m^6^A modification, with each transcript normally containing one to three m^6^A-modified sites [4]. Additionally, the nucleotide fragments resulting from degradation of m^6^A-modified RNA also have a certain signaling effect and can activate the immune response [5]. Taken above, it can be concluded that m^6^A plays important roles in regulating all cell biological processes. Furthermore, m^6^A is involved in necrosis, apoptosis, and autophagy in cells, which can eventually lead to the development of inflammatory diseases [6].

Inflammation is a double-edged sword that is common in various diseases [7, 8]; on the one hand, inflammation fights infection or tissue damage [9, 10], and on the other hand, it could become excessive and cause autologous damage [11]. Recent extensive studies on autoimmune diseases, metabolic diseases, cardio-cerebrovascular diseases, and even cancer have indicated that inflammation contributes more to these diseases [12–15]. Moreover, m^6^A has an essential role in regulating inflammation [16–19]. Recent studies exploring the relationship between m^6^A and inflammation have resulted in novel insights. In this review, we summarize the mechanisms of m^6^A in several inflammatory diseases, as well as its possible role in exploring new therapies from the perspective of epigenetics.

## 2. An Overview of N^6^-Methyladenosine (m^6^A)

**Figure 1:**
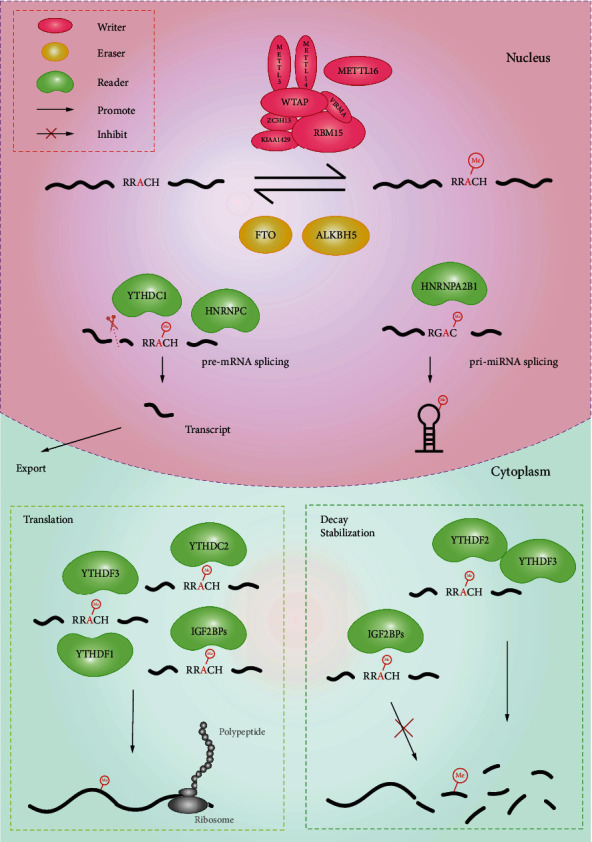
The processes of RNA m^6^A methylation, demethylation, and regulation. m^6^A is read by readers and regulates almost all RNA activities, such as splicing, export, translation, decay, and stabilization.

The participation of methylases (writers), demethylases (erasers), and reader proteins (readers) is required in m^6^A methylation. Methyl is added or removed by writers or erasers to regulate RNA methylation. Readers recognize alterations in methylation and then play regulatory functions in RNA stability, decay, translation, and nuclear output (Figure 1). Finally, m^6^A affects the course of cellular life and influences the occurrence and development of diseases.

### 2.1. Writers

N^6^-Methyladenosine methylation in adenosine is modified by highly conserved RNA methyltransferase complexes, including METTL3, METTL14, WTAP, RBM15, KIAA1429, METTL16, and ZC3H13. METTL3 and METTL14 contain SAM-binding motifs and form stable heterodimers [3, 20]. METTL3 acts as a catalytic subunit, while METTL14 recognizes RNA substrates [21]. At the same time, WTAP and KIAA1429 are responsible for the formation of the complex, and RBM15 is involved in the initial recruitment of the complex to the target site in mRNA [22]. As intensive research on m^6^A has been conducted, more novel methylases have been discovered, such as ZC3H13 and METTL16. Recent studies have shown that ZC3H13 bridges the gap between the aptamers RBM15 and WTAP [23], and METTL16 is an active m^6^A methyltransferase in human cells, which mainly methylates snRNA and intron sites in pre-mRNA [24]. Moreover, two independent studies discovered that rRNA was also subject to m^6^A modification. The METTL5–TRMT112 complex is responsible for human 18S rRNA m^6^A modification [25], while ZCCHC4 is involved in modification of 28S rRNA [26].

### 2.2. Erasers

N^6^-Methyladenosine methylation reversal has been found to be mainly mediated by two demethylases, FTO and ALKBH5. FTO was originally found to be associated with obesity and is the first m^6^A demethylase to be discovered in vitro, in early 2011 [27]. Mechanistically, FTO first oxidizes m^6^A to intermediate N^6^-hydroxymethyladenosine (hm^6^A), which is then converted to N^6^-formyladenosine (f^6^A) and, finally, into adenosine [28]. However, it has been reported that FTO can not only remove m^6^A methylation but also reverse m^6^Am modification with greater efficiency [29]. Shortly after this role of FTO was confirmed, the second mammalian m^6^A demethylase, ALKBH5, was identified [30]. Unlike FTO, ALKBH5 directly converts m^6^A to adenosine in the reverse reaction and without intermediates [31]. Interestingly, both FTO and ALKBH5 are Fe^2+^ and *α*-ketoglutarate-dependent dioxygenase and belong to the ALKBH family; it has been suggested that more ALKBH family proteins or other proteins with similar structures are involved in the demethylation process, resulting in more diverse means for regulating m^6^A methylation. With the development of structural biology, there may be numerous demethylases still waiting to be discovered.

### 2.3. Readers

The m^6^A reader protein is a major player in molecular functions, which mainly includes members of the YTH protein family, IGF2BPs, eIF3, hnRNPs, and Prrc2a. Different reader proteins are distributed in different locations in cells. Nuclear m^6^A readers include YTHDC1 and hnRNPs. Cytoplasmic m^6^A readers include YTHDF1/2/3, YTHDC2, and IGF2BP1/2/3. YTHDC1 recruits splicing factors SRSF3 to regulate the splicing of mRNA [32] as well as affect nuclear output, the decay of specific transcripts [33], and noncoding RNA-mediated gene silencing [34]. hnRNPA2B1 and hnRNPC are also the major reader proteins in the nucleus. hnRNPA2B1 regulated pre-mRNA splicing and promotes primary miRNA processing [35], while hnRNPC only influences pre-mRNA splicing [36, 37]. Moreover, there is a study which indicated that hnRNPG may also be the reader protein of m^6^A [38], which needed to be further confirmed. YTHDC2 mainly affects the translation efficiency of mRNA [39]. YTHDF1 improves translation efficiency by recruiting translation initiation factors in HeLa cells [21, 32]. However, the binding of YTHDF2 to mRNA accelerates mRNA degradation [40]. YTHDF3 regulates the translation or decay of mRNA depending on whether it interacts with YTHDF1 or YTHDF2 [21]. IGF2BPs enhance mRNA stability and translation [41]. Interestingly, IGF2BPs are also able to interact with ncRNA [42], but the regulatory mechanism needs to be further studied [35].

Additionally, eIF3 [43] and Prrc2a [44] are also essential readers. ELAVL1 [45] and G3BPs [46] have been found to repel the binding of m^6^A adenosine to stable mRNA, which may present more interesting competitive functions in the m^6^A reading process.

## 3. Inflammation

Inflammation is the basis of various physiological and pathological processes in humans and animals. Usually, inflammation is an autoadaptive response which is triggered by infection or tissue damage [47, 48]. Furthermore, new interdisciplinary disciplines such as “immune metabolism” have emerged.

### 3.1. Inflammation Initiation

According to the origin of inflammation, inflammatory substances can be divided into two categories: exogenous inducers and endogenous inducers. Exogenous inducers mainly include biological, physical, and chemical factors, while endogenous inducers mainly refer to autoantibodies that induce I–IV autoimmunity and cause varying degrees of inflammation. This in turn leads to the activation of inflammatory mediators.

Inflammatory mediators are molecules that play an important regulatory role in inflammation, and they are mainly derived from blood vessels and cells. Cytogenic inflammatory mediators mainly include vasoactive amines, arachidonic acid metabolites, leukocyte products, cytokines, and platelet activator. Plasma-derived inflammatory mediators mainly include kinin, complement, and the coagulation system.

Generally, inflammation can be classified as acute or chronic based on the course of the inflammation. The degree of inflammation varies according to the stage of a disease. For instance, inflammation is chronic from the beginning in some diseases such as atherosclerosis [49], obesity [50], and cancer [51]. However, acute and chronic inflammation can also coexist, which means that the inflammation is constantly recurring and recovery is difficult, such as in the case of rheumatoid arthritis (RA), multiple sclerosis (MS), and inflammatory bowel disease (IBD). Unfortunately, the mechanisms of these diseases remain unclear [51], but inflammation promotes their progression. Through increased research into inflammation, people have attempted to unravel the molecular mechanisms behind it.

### 3.2. Typical Inflammation Signaling Pathways

**Figure 2:**
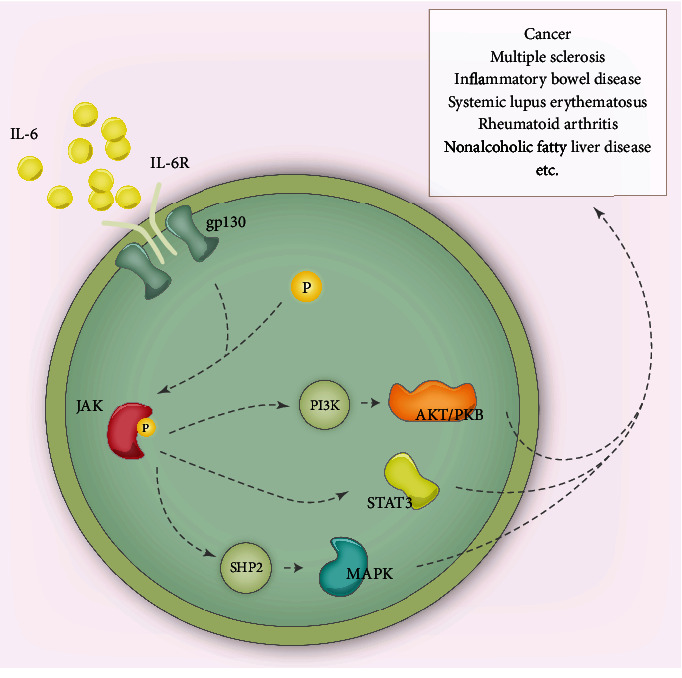
Inflammatory pathways mainly include MAPK pathway, JAK/STAT pathway, and PI3K/AKT pathway.

Abnormal inflammatory responses are being considered as a key factor in human disease. IL-6 is a pleiotropic proinflammatory cytokine, which is an important modular for the transition from acute phase to chronic phase of inflammation. Interestingly, IL-6/IL-6R/gp130 consists of a hexameric complex and activates three essential signaling pathways including MAPK, JAK/STAT3, and PI3K [52] (Figure 2). These pathways are closely associated with cancer, multiple sclerosis, rheumatoid arthritis, inflammatory bowel disease, Crohn's disease, and Alzheimer's disease. In route 1, JAK activates Ras/Raf and subsequently causes hyperphosphorylation of MAPK. In route 2, JAK induces phosphorylation of itself and activates STAT3. Route 3 is associated with the PI3K/PKB/AKT pathway, which contributes to the activation of NF-*κ*B. Finally, multiple inflammatory factors are synthesized by target cells, such as TNF-*α*, IL-1*β*, NO, PGs, IL-6, IL-8, and PAF [9, 53], causing serious tissue damage.

## 4. The Role of m^6^A in Inflammatory Diseases

N^6^-Methyladenosine regulates the expression of inflammation-related mRNAs as well as ncRNA, thereby ultimately regulating inflammatory diseases. Recent studies have shown that m^6^A is closely linked with inflammation (Table 1).

### 4.1. Inflammatory Autoimmune Disease

#### 4.1.1. Multiple Sclerosis

Multiple sclerosis (MS) is a chronic inflammatory neurological disorder that involves demyelinating and neurodegeneration. Typical pathological changes in MS involve scattered and distributed demyelinating plaques around the perivenular inflammatory injury, accompanied by glial fibrosis and axonal injury. There is a wide range of inflammatory infiltrates, mainly including T cells (mainly MHC class 1 restricted CD8+ T cells) and B cells, and oligodendrocyte and macrophages surround the core of a lesion [54].

**Figure 3:**
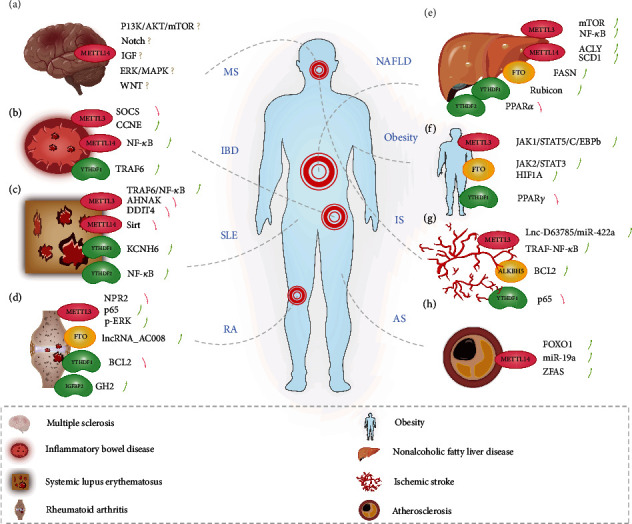
The role of m6A in (a–d) inflammatory autoimmune diseases, (e, f) metabolic disorder, and (g, h) cardio-cerebrovascular diseases: (a) multiple sclerosis (MS); (b) inflammatory bowel disease (IBD); (c) systemic lupus erythematosus (SLE); (d) rheumatoid arthritis (RA); (e) obesity; (f) nonalcoholic fatty liver disease (NAFLD); (g) ischemic stroke (IS); (h) atherosclerosis (AS).

Recently, studies have shown the modification of m^6^A methylation in cerebrospinal fluid that involves the development of multiple sclerosis. It has been reported that patients who suffered from MS usually have higher m^6^A methylase expression [55]. In addition, the m^6^A and expression levels of inflammation-related mRNA in the patients with relapsing remitting multiple sclerosis (RRMS) are significantly higher than those in progressive multiple sclerosis (PMS) [56]. To explore the role of m^6^A in nerve cells, Xu et al. [57] found that knocking out METTL14 in oligodendrocytes leads to hypomethylation of the mRNA of several transcription factors, growth factors, and histone modifiers. This disrupts the normal maturation and differentiation of oligodendrocytes and led to abnormal splicing of large amounts of transcripts that are significantly enriched in several inflammatory signaling pathways, such as the PI3K/AKT/mTOR, ERK/MAPK, IGF-1, Notch, and WNT signaling pathways [57] (Figure 3(a)).

#### 4.1.2. Inflammatory Bowel Disease

Inflammatory bowel disease (IBD) is characterized by atopic chronic inflammation that is often associated with a variety of factors, such as food, the environment, and heredity [58], and includes ulcerative colitis (UC) and Crohn's disease (CD) [59].

Numerous studies have shown an association between m^6^A and the mucosal immune microenvironment [16]; therefore, scholars have speculated that m^6^A also plays a critical role in IBD. By testing the clinical samples of CRC, researchers have found that METTL3 promotes cell proliferation through suppressing SOCS2 [60] and stabilizing CCNE1 in an m^6^A-dependent manner [61]. Also, METTL14 is essential for suppressing apoptosis in colonic epithelial cells through the NF-*κ*B pathway [62]. The lack of METTL14 in T cells has been shown to induce spontaneous colitis in mice and is accompanied by severe inflammatory cell infiltration [63]. Therefore, abnormal cell proliferation, chronic inflammation, and antiapoptotic processes occur in intestinal cells where hypermethylation is observed, which may further aggravate IBD. Additionally, YTHDF1 promotes the expression of TRAF6 [64], and YTHDF1 knockout strongly inhibits WNT-driven regeneration and tumorigenesis [65, 66].

Recently, a comprehensive analysis of m^6^A in IBD was carried out. IGF2BP2, HNRNPA2B1, ZCCHC4, and EIF3I showed significantly different expression patterns in colon biopsy samples of patients with IBD [67]. Sebastian-delaCruz et al. [68] predicted that m^6^A also regulates genes associated with IBD, such as UBE2L3 and SLC22A4 in Crohn's disease and TCF19, C6orf47, and SNAPC4 in ulcerative colitis. High expression of m^6^A-related phenotype genes, such as H2AFZ, is often accompanied by higher abundances of M1 macrophages, M0 macrophages, and naive B cells in IBD patients. This research has guided drug selection in the direction of m^6^A and provided ideas for improving responses to anti-TNF treatment [16]. However, experiments that confirm the diagnostic significance or therapeutic value of the m^6^A regulatory gene in IBD are lacking, and further research is still needed (Figure 3(b)).

#### 4.1.3. Systemic Lupus Erythematosus

Systemic lupus erythematosus (SLE) is a typical multisystem inflammatory autoimmune disease [69]. Normally, lupus nephritis (LN) is one of the most common severe organ manifestations of SLE, which is related to high mortality [70]. Neuropsychiatric lupus (NPSLE) occurs in 40–90% of SLE patients, and the damage of microglia may be the major damaged cell [71–73]. Actually, NPSLE is a major source of morbidity in the SLE population, and its mortality is second only to that of LN.

Single-cell transcriptomics analysis has shown that microglia in mice exhibit upregulation of multiple inflammatory genes, for which m^6^A plays an important role [74]. To explore the m^6^A mechanism of cell injury in SLE, researchers have found that METTL3 is an important writer that promotes LPS-induced microglia inflammation through the TRAF6/NF-*κ*B pathway [75]. Moreover, METTL3 has been found to regulate the repair of corneal cell damage [76], while METTL14 exacerbates the progression of renal epithelial cell damage by downregulating Sirt1 [77]. m^6^A is supposed to be the protective factor in SLE. Other studies also confirmed that the expression levels of METTL3, METTL14, WTAP, FTO, ALKBH5, and YTHDF3 in patients with SLE are significantly downregulated [78, 79]. Downregulation of ALKBH5 in peripheral blood may be related to the pathogenesis of SLE, and scientists have observed a strong correlation between ALKBH5 expression and patient autoantibody levels as well as clinical features [78].

Studies have shown that YTHDF1 regulates KCNH6 in an m^6^A-dependent manner and affects the transition from lung fibroblasts to myofibroblasts [80]. YTHDF2 regulates the CircGARS–miR-19a–TNFAIP3 axis through a sponge mechanism, which mediates immune activation of NF-*κ*B and ultimately promotes SLE progression [81]. IGFBP3 is an important biological marker of SLE disease, and it may indirectly inhibit the immune response by reducing the regulation of T cell and B cell activities, thus achieving therapeutic effects [82]. Interestingly, these studies may provide preliminary evidence for taking an epigenetic perspective in SLE targeted therapies, but there is still an urgent need for more detailed reports with clear evidence supporting targeting of m^6^A in SLE therapy (Figure 3(c)).

#### 4.1.4. Rheumatoid Arthritis

Rheumatoid arthritis (RA) is a systemic disease primarily dominated by synovial inflammation that results in synovitis, synovial hypertrophy, and cartilage/bone destruction [83]. Eventual disease progression leads to multiorgan inflammatory damage, affecting the skin, lungs, heart, and/or eyes [83–85] with the development of the course of disease. However, the mechanism of RA is still unclear, and most researchers believe that this autoimmune disease is the result of a combination of epigenetic and genetic factors recently.

Based on single-cell sequencing and machine learning methods, some researchers have identified neuropeptide-related molecules with key regulatory activities in RA, and they have suggested that METTL3 downregulation and IGF2BP2 upregulation aggravate RA through GHR and NPR2 [19]. METTL3 knockdown decreased the percentage of apoptosis and, through the NF-*κ*B pathway, the expression of inflammatory factors in chondrocytes that had been induced by IL-1*β* [86]. Furthermore, recent studies have shown that reductions in ALKBH5, FTO, and YTHDF2 may be key risk factors for patients who suffer from RA [87]. Moreover, m^6^A-modified noncoding RNA can also regulate the inflammatory response of chondrocytes, for instance, lncRNA_AC008 accelerates the exacerbation of inflammatory damage in chondrocytes through the miR-328-3p-AQP1/ANKH axis under the regulation of FTO [88]. Another important gene, BCL2, regulates chondrocyte' apoptosis and autophagy in an YTHDF1-dependent manner [89].

Interestingly, *Sarsasapogenin*, as a representative anti-inflammatory traditional medicine, acts on the m^6^A methylation-modified gene TGM2 in the immune microenvironment of synovial tissue, regulating the cell cycle and reducing apoptosis, which can effectively alleviate the development of RA diseases [90]. However, as a global autoimmune disease, there is still no clear explanation of the actual mechanism of RA. It is hoped that studies on m^6^A could provide us with new directions for studying this disease (Figure 3(d)).

### 4.2. Inflammatory Metabolic Disorder

#### 4.2.1. Nonalcoholic Fatty Liver Disease

Nonalcoholic fatty liver disease (NAFLD) is one of the most common causes of chronic liver disease, with a global prevalence of about 25% [91]. Mechanically, NAFLD is usually accompanied by inflammation and liver fibrosis [92], which progresses to hepatocellular carcinoma in severe cases.

METTL3 regulates hepatocyte ploidy, and METTL3 knockout results in global hypomethylation, which leads to a series of pathological features associated with NAFLD (e.g., hepatocyte ballooning, microsteatosis, polymorphic nucleus, and DNA damage) [93]. Knocking out METTL3 can effectively inhibit the mTOR and NF-*κ*B signaling pathways, alleviating NAFLD and inflammation in mice [94]. Moreover, METTL3 and METTL14 have been shown to affect triglyceride and cholesterol production and lipid droplet accumulation through ACLY and SCD1 in vitro [95].

Since NAFLD and lipid metabolism are inextricably linked, FTO has also attracted attention in the study of NAFLD, where it reduces mitochondrial abundance and promotes hepatic fat accumulation in hepatocytes, which are hypomethylated [96]. Recent research has explored FTO-regulated hepatic lipid production through FASN. Knockdown of FTO decreased the expression of FASN, which inhibited de novo lipogenesis, thereby resulting in deficient lipid accumulation and induction of cellular apoptosis [97]. Hypermethylated rubicon mRNA is expressed and bound by YTHDF1, which increases its stability, with autophagy in the liver being ultimately inhibited, which leads to accumulation of lipid droplets [98]. YTHDF2 binds to PPAR*α*, leading to alterations in the circadian rhythm, and mRNA stability is mediated to regulate lipid metabolism [99] (Figure 3(e)).

#### 4.2.2. Obesity

Maintenance of homeostasis is essential to the proper operation of the body's life activities, and extensive inflammation and stress occur when the body's metabolism is disturbed. In obesity, excess adipose tissue can secrete more adipokines, such as leptin and interleukin. Excess interleukin promotes adipose tissue infiltration of immune cells, which leads to chronic low-grade inflammation and promotes insulin resistance [100, 101].

In a recent study, the demethylation activity of FTO was shown to be necessary for preadipocyte differentiation [102] and fat metabolism [103], and FTO was identified as an important bridge between obesity and m^6^A. FTO reduces apoptosis of fat cells by activating the JAK2/STAT3 signaling pathway [104]. Zfp217 activates FTO through interaction with YTHDF2 and preserves adipose differentiation [105]. Another study indicated that white-to-beige fat transition is promoted by HIF1A with hypermethylation mediated by FTO [106]. However, some researchers have reported that m^6^Am, instead of m^6^A, was the substrate of FTO. As FTO expression increased, the fatty acid-binding proteins FABP2 and FABP5 lost their m^6^Am modification, and their expression was downregulated [107]. Above all, characterizing the role of the FTO gene has greatly contributed to clarifying the mechanism of m^6^A in obesity.

With more in-depth research on obesity, more relationships between m^6^A and obesity have been discovered. METTL3 was also found to be an essential regulatory protein in obesity [108]. A recent study found that METTL3 inhibits adipocyte differentiation through the JAK1/STAT5/C/EBP*β* pathway [109]. Additionally, WTAP and METTL14 have also been found to affect the differentiation of adipocytes [110]. Moreover, two SNPs in METTL3 were found to be associated with body mass index (BMI), and two SNPs in YTHDF3 are associated with gene expression [17]. Clinical studies have explored that curcumin promotes TRAF4 m^6^A methylation and its expression levels under the mediation of YTHDF1, which finally promotes PPAR*γ* degradation through the ubiquitin proteasome pathway, thereby inhibiting lipogenesis [111]. However, details of the complex mechanisms by which m^6^A regulates obesity remain unclear (Figure 3(f)).

### 4.3. Inflammatory Cardio-Cerebrovascular Diseases

#### 4.3.1. Ischemic Stroke

Ischemic stroke (IS) is one of the most harmful cardio-cerebrovascular diseases. Due to acute ischemia of brain tissue, nerve cells are hypoperfused, leading to local depletion of oxygen and glucose [112]. The infarction area undergoes the death of nerve cells, during which apoptosis and autophagy are the main pathological features [113–115].

Ischemia was first thought to be the only factor to cause tissue damage. However, through extensive review of cases of ischemic diseases, such as stroke and renal ischemic renal failure, scientists gradually discovered that ischemic reperfusion damage leads to more serious damage to tissues. The source of damage is mainly from free radicals and inflammation. Venous recombinant tissue plasminogen activators (rt-PA) are currently proven treatments for stroke, but they are only effective within three hours of a limited onset [116]. Therefore, there is an urgent need to explore new directions for treating IS. In an m^6^A transcriptome-wide map of an MCAO mouse model, 17 lncRNAs and 22 mRNAs with hypermethylation and 5 mRNAs and 3 lncRNAs with hypomethylation were found. The function of these altered m^6^A transcripts was found to be mainly enriched in inflammation, apoptosis, and brain damage [117]. They found that the expression of inflammatory cytokines (IL-1*β*, IL-6, TNF-*α*, and IL-18) and inflammatory enzymes (TRAF6 and NF-*κ*B) is upregulated as the expression of METTL3 increases in microglia inflammation. Also, overexpression of METTL3 promotes activation of the TRAF6-NF-*κ*B pathway in an m^6^A-dependent manner, and it inhibits inflammation [75]. Another study showed that in the I/R model, the overall m^6^A methylation level was upregulated, and there were significant differences in METTL3, FTO, and ALKBH5 [118, 119]. In a recent study, an oxygen glucose deprivation/reoxygenation (OGD/R) model was established successfully by inducing nerve cell injury, and the expression of Lnc-D63785 with hypermethylation decreased in a METTL3-dependent manner, which led to the accumulation of miR-422a and resulted in cell apoptosis in primary murine neurons [120]. Another study also showed that knocking down ALKBH5 can aggravate neuronal damage and demethylases ALKBH5/FTO coregulate m6A demethylation, resulting in neuronal apoptosis mediated by BCL-2 [118]. Therefore, it is speculated that demethylase also has a protective role in I/R damage and in preventing reperfusion damage [117]. This lays the foundation for the clinical application of m^6^A modification. miR-421-3p specifically targets YTHDF1 and inhibits translation of p65, suggesting a possible role for m^6^A in IS [121]. There is a complex regulatory network precisely regulating our circulatory system based on the extensive role of m^6^A methylation (Figure 3(g)).

#### 4.3.2. Atherosclerosis

Atherosclerosis (AS) occurs due to the accumulation of cholesterol in vessel walls, and recent research has suggested that chronic inflammation may be the real cause of AS [122]. Arteries are divided into three layers, of which the innermost intima mainly consists of collagen fibers, elastin fibers, and a small number of smooth muscle cells. Under homeostatic conditions, endothelial monolayers do not attract the aggregation of leukocytes and lead to immune activation. When inflammatory cytokines or other cardiovascular risk factors are present, endothelial cells promote the adhesion of immune cells and induce inflammation [123].

HSP60 and LDL act as antigens in the development of the disease, causing cellular immunity and humoral immunity [124], while CD4^+^ T cells handle these antigens [125]. Study revealed that METTL14 targets mir-19a and facilitated the treatment of mature miR-19a, thus promoting the proliferation and invasion of atherosclerotic vascular endothelial cells [126]. Hypermethylation may be a risk factor for aggravating the inflammation of AS.

AS is the most extensive cardiovascular disease currently known, and surveys have shown that patients with other chronic inflammation disorders have a higher probability of developing AS [127]. Recently, a study showed that the expression of ZFAS1 with hypermethylation increases under the regulation of METTL14 [128], which activates the downstream ADAM10/RAB22A pathway in an epigenetic modification manner, and ultimately participates in the inflammatory process of vascular endothelial cells in AS [129]. In fact, numerous reports have revealed that AS is regulated by m^6^A modification, mainly based on inflammatory models of endothelial cells [126, 130], macrophages [131, 132], and smooth muscle cells [133, 134]. Although these studies aim to simulate what happens to patients with AS, they cannot truly reflect the conditions of AS patients. More clinical research is needed to explain the epigenetic mechanisms of AS pathogenesis (Figure 3(h)).

## 5. The Role of m^6^A in Inflammatory Cancer Microenvironments

Cancer has always been a difficult problem in the medical community. Extensive studies have showed that inflammatory microenvironments are associated with cancer progression or inhibition [135–139], and m^6^A provides new ideas for clinical diagnosis and treatment. Globally, liver cancer is the most frequent fatal malignancy [140]. Lung cancer is one of the most common malignant tumors and is the most frequently diagnosed fatal tumor type in China [141].

**Figure 4:**
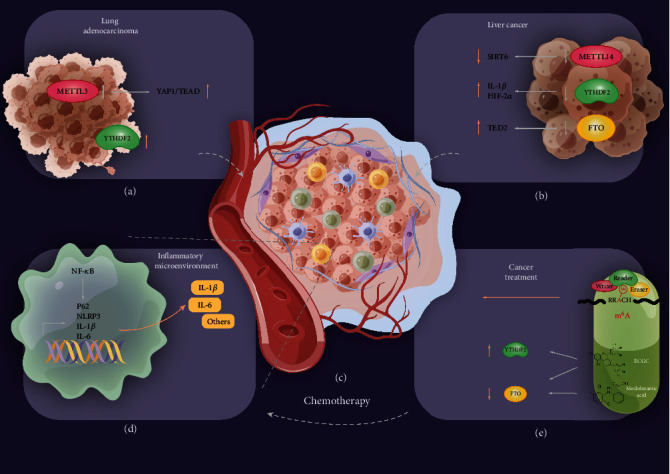
Cancer is regulated by m^6^A and the inflammatory microenvironment. In (a) lung adenocarcinoma and (b) human liver cancer, m^6^A modification regulates the expression of the target inflammatory gene. (c) Microenvironment inside the cancer and (d) numerous cytokines, such as IL-1*β* and IL-6, modulate the inflammatory microenvironment to stimulate tumor growth and invasion. (e) m^6^A application and targeting therapy in cancer through the inflammatory microenvironment.

In human liver cancer, the expression of USP48 is downregulated, reducing the stability of SIRT6, which induces the occurrence of liver cancer. Recent studies have shown that METTL14 is involved in the stabilization of USP48, thereby hindering the occurrence of liver cancer tumors [142]. YTHDF2, as an important reading protein, is significantly downregulated in liver cancer cells and leads to severe inflammation, vascular reconstruction, and cancer metastasis. The deletion of YTHDF2 causes mRNA decay of IL-1*β* and HIF-2*α*. After using an HIF-2*α* inhibitor, the authors found that liver cancer was suppressed [143]. Additionally, FTO can also inhibit tumor growth by reducing TED2 mRNA stability [144] (Figure 4(a)).

Recently, researchers again confirmed that IL-6 can effectively construct the inflammatory microenvironment of lung adenocarcinoma liver metastasis in vitro, increasing the proliferation, metastasis, and EMT of lung adenocarcinoma cells [145–149]. Moreover, the authors found global RNA methylation increases and METTL3 activates the YAP1/TEAD signaling pathway. Another study showed that high expression of YTHDF2 and SUMO1 often suggests a poor prognosis for lung adenocarcinoma. Sumoylation of YTHDF2 can increase its ability to bind to m^6^A-modified mRNA to downregulate target gene expression, ultimately inducing cancer [150] (Figure 4(b)).

The relationship between cancer and inflammation is not a new idea, and as early as 1863, Verchow proposed the hypothesis that cancer may originate from inflammation [6]. Studies have documented that in the inflammatory microenvironment (Figure 4(c)), where inflammatory factors such as IL-1*β* and IL-6 are enriched (Figure 4(d)), tumor cells are more likely to proliferate, metastasize, and perform EMT [151]. The use of aspirin and nonsteroidal anti-inflammatory drugs (NSAIDs) can effectively reduce the risk of several types of cancer by 40–50%, such as colon cancer, which demonstrates the important impact of inflammation in the cancer process. Immunotherapy against the tumor inflammatory microenvironment has a significant curative effect on tumors with activation of inflammatory pathways, infiltration of active immune cells, and lack of matrix components [152]. Researchers have found that writers and erasers regulate malignant tumors in a reading protein-dependent manner, and they are mainly concentrated in inflammatory pathways [152, 153]. This provides new ideas for personalized treatment of tumors. For instance, IFITM3, as an important innate immune protein, may be associated with the microbiota and m^6^A. At the same time, IFITM3 is positively correlated with immunomodulators, tumor-infiltrating immune cells (TIIC), and cancer immune cycles [154].

In clinic, chemotherapy is a common cancer treatment (Figure 4(e)). Aseptic inflammation caused by chemotherapy is a serious negative effect, while ALKBH5 affects the progression of aseptic inflammation through epidermal modifications [155]. Above all, m^6^A plays an important role in not only the development of cancer but also in controlling the negative effects of chemotherapy. m^6^A may be a crucial mechanism in cancer, and further epigenetics research into cancer could lead to further breakthroughs in cancer treatments. Moreover, numerous preclinical studies indicated that m^6^A targeting therapy synchronizing with anti-PD1 therapy has shown tremendous potential.

## 6. The Role of m^6^A in Pathogen-Induced Inflammation

Extensive studies have shown that m^6^A plays a wide range of roles in pathogen-induced inflammation. METTL3 has attracted considerable attention. Overexpression of METTL3 can significantly reduce LPS-induced inflammation in macrophage [156]. METTL3 depletion inhibits YTHDF1- and YTHDF2-mediated degradation of NOD1 and RIPK2, which subsequently promotes LPS-induced inflammatory responses [157]. Another research demonstrated that LPS stimulation leads to upregulation of YTHDF2, and knockdown of YTHDF2 improves the stability of MAP2K4 and MAP4K4 and activates the MAPK and NF-*κ*B signaling pathways [131].

In *Salmonella typhimurium* infection, YTHDF2 depletion promoted H3K27me3 demethylation of multiple inflammatory cytokines in the MAPK and NF-*κ*B signaling pathways, such as IL-6 and IL-12B, and subsequently enhanced its transcription [158]. Moreover, cell injury induced by *E. coli* and *S. aureus* has also been proved to be associated with m^6^A modification, and differentially expressed genes are mainly enriched in inflammation, apoptosis, and autophagy [47, 159].

The role of m^6^A modification has also been found in fungi-induced inflammation. *Fusarium solani*-induced keratitis increased global m^6^A level and the expression of METTL3 in corneal stromal cell and mice, which ultimately activated the NF-*κ*B signaling pathway [160].

Recently, researchers showed that METTL3 depletion in host cells simultaneously reduced m^6^A levels in both the host and SARS-CoV-2. The global hypomethylation in SARS-CoV-2 increased RIG-I and enhanced the expression of innate immune signaling pathways and inflammatory genes relatedly [161]. This may indicate that SARS-CoV-2 undergoes m^6^A modification through host m^6^A machinery and regulates its own activities [162].

Totally, it is widely acknowledged that m^6^A modification plays important roles in pathogen-induced inflammation.

## 7. Clinical Therapy Potential

Taken together, m^6^A has clinical therapeutic potential for the treatment of inflammatory disease. Recently, studies have discovered highly effective compounds that target m^6^A modification [163–165]. For instance, meclofenamic acid (MA) was directly identified as a specific inhibitor of FTO through screening [166]. The natural compound radicicol was proved to be a potent FTO inhibitor [167]. STM2457 is a highly potent and selective first-in-class catalytic inhibitor of METTL3, which can reduce acute myeloid leukemia (AML) growth [164]. Moreover, traditional medicines have also been found to be important m^6^A modulators [168–171]. Resveratrol and curcumin are natural phenolic compounds that increase YTHDF2 levels to maintain intestinal mucosal integrity, which has potential for IBD treatment [111, 172, 173]. Epigallocatechin gallate is a tea flavonoid, and it exerts a strong anti-inflammatory effect mainly by inhibiting FTO expression and enhancing YTHDF2 expression [174]. Saikosaponin, an extract of Bupleuri, also has anti-inflammatory activity, inhibiting FTO expression [175]. With the development of modern medicine, photoactivated compounds have been creatively constructed, such as a caged molecule activator of METTL3/14, photocaging substituent-linked MPCH [176]. This drug can be rapidly released by exogenous light and functions in vivo, and it is considered a breakthrough in m^6^A-targeted drugs, though its side effects still need to be strictly monitored.

## 8. Future Prospects

Recently, many studies have shown that m^6^A is closely related to inflammation and can thus be considered a target for treatment. As an epigenetic modification found in various RNAs, m^6^A in ncRNA can represent a more precise target than those used in traditional medicine. With the development of high-throughput sequencing technology and MeRIP-seq, used in combination with GWAS and SNP analyses, it may be possible to gain more biological information about m^6^A modifications. Comprehensive studies of m^6^A have been beneficial for enhancing our understanding of inflammation.

Moreover, it is more efficient to develop m^6^A agonists or inhibitors for treating inflammation by characterizing the m^6^A profiles in different diseases, which could replace some traditional drugs that have extensive side effects, such as corticosteroids. In summary, m^6^A has the potential to become a prospective target in treatments for inflammation, but further confirmation is still needed.

## Figures and Tables

**Table 1:** The role of m^6^A in inflammatory diseases.

m^6^A component		Target gene	Inflammatory mechanism	Cell/cell line	Diseases	Reference
Writer	METTL3	TRAF6	TRAF6/NF-*κ*B pathway	Microglia	Microglia inflammation	[75]
		RIG-I	NF-*κ*B pathway	Caco-2 Calu-3 HEK293FT	SARS	[161]
		PGC-1*α* STAT1	STAT1 signaling pathway	THP-1 HEK293T HUVECs RAW264.7	Mononuclear-macrophage inflammation	[177, 178]
		hsa_circ_0029589	IRF1/hsa_circ_0029589 axis	Macrophages	Atherosclerosis	[132]
		ATG7	—	Fibroblast-like synoviocytes ATDC5	Osteoarthritis	[86, 179]
		MyD88	NF-*κ*B and MAPK signaling pathways	HDPCs	Pulpitis	[180]
		miR-21-5p	SPRY1/ERK/NF-*κ*B axis	HK-2	Nephritis	[181]
		TIMP2	Notch3/4 pathway	MPC5 mTECs	Diabetic nephropathy	[179]
		p65, p-ERK, MMP-1, MMP-3	NF-*κ*B and MAPK signaling pathways	SW1353	Rheumatoid arthritis	[182]
		STAT2	SNHG4/STAT2 axis	WI-38	Fibrous pneumonia	[183]
		NF-*κ*B	NF-*κ*B pathway	MODE-K	Inflammatory bowel disease	[184]
	METTL14	FOXO1	VCAM-1/ICAM-1 transcription	HUVECs THP-1 HEK293T	Atherosclerosis	[130]
		ELMO1	—	MSCs HEK293T	Ankylosing spondylitis	[185]
		*α*-klotho, Sirt1	—	HRGECs Podocytes	Nephritis, diabetic	[77, 186]
		NFASC	—	Oligodendrocyte	Multiple sclerosis	[57]
	WTAP	RIPK2, JAK3, TNFRSF10A	Inflammatory-related pathways	MH7A	Rheumatoid arthritis	[187]

Eraser	FTO	IL-6, TNF-*α*, IL-1*β*	Inflammatory factor signaling pathway	H9c2	Myocarditis, endotoxemia	[188]
		YAP1	Cell apoptosis and inflammation	Cardiomyocytes	Ischemia-reperfusion injury	[18]
	ALKBH5	AC008440.5	AC008-miR-328-3p-AQP1/ANKH axis	Articular chondrocytes, HEK293T	Osteoarthritis	[88]
		IL-6	—	THP1, BEAS-2B	Radiation aseptic inflammation	[155]
		HMGB1	STING/IRF3 axis	HSCs	Radiation hepatitis	[189]

Reader	IGF2BP2	C/EBPs	—	MEFs, HK-2, HEK293T	Multiple sclerosis, glomerulonephritis	[190]
		TSC1	Macrophage M1 differentiate to M2	BMDMs, macrophage	Ulcerative colitis, allergic pneumonia, asthma	[191]
	IGF2BP3	hsa_circ_0004287	p38/MAPK pathway	THP1, Raw264.7, HACAT, PBMC	Atopic dermatitis	[192]
	YTHDF1	P65	YTHDF1/miR-421-3p/p65 axis	BV2	Ischemic stroke	[121]
		XPO1	NF-*κ*B pathway	HCT116, C26, human Jurkat T cell line	Celiac disease	[193]
	YTHDF2	KDM6B	Demethylation of H3K27me3	THP-1, HEK293T, PBMCs	Bacterial infectious inflammation	[158]
		IFN, TLR4, STAT1, IRF7, TNF	Multiple inflammatory pathways	Splenic cells	Acute myeloid leukemia	[194]

Note: the horizontal line represents undetermined. Caco-2 cells: human colorectal adenocarcinoma cell lines; Calu-3: human lung adenocarcinoma cell line; HEK 293T and HEK293FT: human embryonic kidney cell line; THP-1 cells: human peripheral blood monocyte cell line; HRGECs: human renal glomerular endothelial cells; H9c2 cells: rat cardiomyocytes cell line; HUVECs: human umbilical vein endothelial cells; RAW264.7 cells: murine macrophage cell line; ATDC-5 cells: murine chondrocyte cell line; HDPCs: human dental pulp cells; HK-2 cells: human proximal tubule epithelial cell line; MPC5 cells: mouse podocytes; mTECs: medullary thymic epithelial cells; WI-38 cells: human lung fibroblast cells; MODE-K cells: murine duodenal epithelial cells; MSCs: mesenchymal stem cells; BEAS-2B cells: human normal lung epithelial cells; HSCs: hematopoietic stem cells; MEFs: mouse embryonic fibroblasts; BMDMs: murine bone marrow-derived macrophages; HACAT: human keratinocyte cell line; PBMCs: peripheral blood mononuclear cells.

## Abbreviations

m^6^A: N^6^-Methyladenosine
MS: Multiple sclerosis
RRMS: Relapsed-remission multiple sclerosis
PMS: Progressive multiple sclerosis
IBD: Inflammatory bowel disease
UC: Ulcerative colitis
CD: Crohn's disease
SLE: Systemic lupus erythematosus
RA: Rheumatoid arthritis
NAFLD: Nonalcoholic fatty liver disease
MeRIP-seq: Methylated RNA immunoprecipitation sequencing
GWAS: Genome-wide association study
SNP: Single nucleotide polymorphism
LN: Lupus nephritis
NPSLE: Neuropsychiatric lupus
MA: Meclofenamic acid
AML: Acute myeloid leukemia.
